# LANCL1 as the Key Immune Marker in Neuropathic Pain

**DOI:** 10.1155/2022/9762244

**Published:** 2022-04-25

**Authors:** Yu Shi, XueFei Zhang, Qian Fang, Hongrui Zhan, Xianglong Wang, Xiyan Huang, Tao Fan, Wei Liu, Wen Wu

**Affiliations:** ^1^Department of Rehabilitation, Zhujiang Hospital, Southern Medical University, Guangzhou 510282, China; ^2^Department of Rehabilitation, The Fifth Affiliated Hospital of Sun Yat-sen University, Zhuhai 519000, China; ^3^Department of Rehabilitation, Guangzhou Red Cross Hospital, Jinan University, Guangzhou 510000, China

## Abstract

**Objective:**

This study is to explore key immune markers and changes of immune microenvironment in neuropathic pain (NeuP).

**Method:**

The data sets of GSE145199 and GSE145226 in Gene Expression Omnibus (GEO) database was used to analyze, and the key immune markers were verified by GSE70006 and GSE91396, and the infiltration degree of immune cells in different samples were analyzed by CIBERSORT analysis package.

**Results:**

In this study, we found a key immune marker, namely, LANCL1. Regulatory axis closely related to LANCL1 has also been found, namely, miR-6325/LANCL1 axis. In the immune infiltration analysis, we also found that the LANCL1 is positively correlated with T cells CD4 naïve (*r* = 0.880, *p* < 0.05).

**Conclusion:**

In this study, we found that LANCL1 may be a protective factor for NeuP, and the miR-6325/LANCL1 axis may be involved in the occurrence and development of NeuP. Cascade reactions including mast cells, macrophages, and T cells may be an important reason for the aggravation of nerve damage.

## 1. Introduction

Neuropathic pain (NeuP) is a type of pain caused by injury or disease of the nervous system. Its clinical manifestations are hyperalgesia, paresthesia, and spontaneous pain. It is often complicated with sleep disorders, depression, and anxiety [1, 2]. It is estimated that at least 1% -5% of the population suffer from NeuP throughout the year [3]. Because of the diversity of pathogenic factors and the complex pathological mechanism in NeuP, the clinical treatment effect is not satisfactory, which can cause patients to appear serious physiological and psychological disorders, and seriously reduce the quality of life [4, 5]. Therefore, it is of great significance to explore the pathogenesis and prevention of NeuP. Current studies suggest that, imbalance between excitatory and inhibitory somatosensory signals [6], changes in ion channels [7], and variability of pain signals in the central nervous system all have been related with the NeuP. However, the above mechanisms cannot fully explain the occurrence and development of NeuP, and further exploration is needed.

In recent years, researchers have suggested that inflammation and immune mechanisms in the peripheral and central nervous systems play an important role in NeuP [8]. Infiltration of inflammatory cells and activation of innate immune cells activated in response to nervous system damage lead to subsequent production and secretion of various inflammatory mediators. These mediators promote neuroimmune activation and can sensitize primary afferent neurons and cause hypersensitivity to pain [9]. It is well known that nerve injury leads to activation of mast cells and recruitment of neutrophils and macrophages. Tumor necrosis factor (TNF) and interleukin 1 and 6 (IL-1, IL-6) released by immune cells are believed to be closely related to hyperalgesia of NeuP and play an important role in the occurrence and development of NeuP [10]. Some of the molecules expressed in gene translation are closely related to immune infiltration and are defined as immune genes [11]. Several immune genes or immune molecules have been shown to play a role in NeuP, such as miRNA-23a/CXCR4 axis [12] and miR-136/IL6R axis [13]. At present, the research on the pathway mechanism of NeuP mainly relies on the verification analysis of the discovered factors, and there is still a lack of screening for the key immune markers of NeuP, leading to the possible omission of the signal axis. Therefore, in order to obtain immune factors closely related to NeuP, we included all immune genes that had been confirmed in the previous studies [14] for screening. At the same time, we also included immune infiltration analysis to obtain the infiltration degree of immune cells in different samples to understand the changes in the immune microenvironment of NeuP. It is of great significance to explore the role of key immune markers in immune infiltration and the changes of immune microenvironment from the perspective of immune cell infiltration to reveal the mechanism of NeuP. CIBERSORT [15] is based on immune infiltration data, which allows the use of a transcriptome expression matrix to estimate the abundance of immune cells and other stromal cells in tissue infiltration. CIBERSORT was first used in the analysis of cancer-associated immune infiltration [16] and is now being used in other immune-related studies of nontumor inflammatory responses [17].

In this study, in order to improve the reliability of the research results, we analyzed and estimated mRNA and miRNA data sets sequenced from the same sample set from the Gene Expression Omnibus (GEO) database. Previous studies have shown that neuropathic pain behaviors correlate with synaptic plasticity and limbic cortex alteration [18]. However, the previous screening of key markers of neuropathic pain mostly focused on the inflammatory changes of spinal cord neurons and paid less attention to the inflammatory changes of limbic system. Therefore, we selected the gene data set from limbic cortex for exploration. Key immune markers were obtained through immune gene extraction, differential gene analysis, least absolute shrinkage and selection operator (LASSO) regression model [19] screening, and receiver operating characteristic (ROC) analysis verification. Correlation analysis of mRNAs (key immune markers) and miRNAs was then performed, and online databases (mirWalk and TargetScan) were used to predict the miRNAs likely to bind to key immune markers, and the key miRNA/mRNA signaling axis were obtained after the intersection. Finally, the CIBERSORT analysis package was used to analyze the degree of immune cell infiltration in matrix data and obtain the correlation information between the key immune markers and the immune infiltrated cells. Through this research, we hope to obtain the key immune markers of NeuP and increase the understanding of the immune microenvironment changes in NeuP, so as to provide ideas and help for future research.

## 2. Methods

### 2.1. Data Source

In this study, we used the GSE145199 data set (miRNA) [20] and GSE145226 data set (mRNA) [20] in the GEO database as the training set for estimation and analysis and used the data sets GSE70006 and GSE91396 as the verification set to verify the results.

### 2.2. Data Preprocessing

In this step, we used the R (V4.0.4) software (https://www.r-project.org/) to preprocess the data, including correction and normalization.

### 2.3. Immune Gene Extraction

In this step, we used the immune gene data provided in the online database ImmPort [14] (https://www.immport.org) to extract the matrix data.

### 2.4. Differential Expressed Genes (DEGs) Analysis

In this step, we used limma analysis package [21] to perform differential gene analysis on the immune gene data matrix. DEGs with *p* < 0.05 and |log2FC| >0.5 were considered statistically significant. Then, we used the impute [22] and pheatmap [23] analysis packages to draw the volcano map and heatmap of the DEGs.

### 2.5. LASSO Analysis Screen

In this step, we performed LASSO regression analysis on immune genomic matrix data using the glmnet [24] analysis package to screen for hubgenes that may be closely associated with NeuP. After that, we intersected the screened hubgenes with DEGs to obtain the key immune markers. And the online website bioinformatics (https://www.bioinformatics.com.cn) was used to draw the Venn diagram.

### 2.6. ROC Verification of Key Immune Markers

In this step, we used the bioinformatics to perform ROC analysis and draw ROC curve in the verification data sets of GSE70006 [25] and GSE91396 [26]. Hubgenes with *p* < 0.05 and AUC > 0.7 were considered statistically significant; these hubgenes were considered to be key gene markers.

### 2.7. Correlation Analysis of mRNAs (Key Immune Markers) and miRNAs

In this step, we used the reshape2 [27], dplyr (https://dplyr.tidyverse.org/), and tidyr (https://tidyr.tidyverse.org) analysis packages for the correlation analysis between mRNA and miRNA. According to the binding regulation principle of mRNA and miRNA, correlations with correlation coefficient < -0.4 and *p* < 0.05 were considered statistically significant.

### 2.8. miRNA Prediction

In this step, we used the online website mirWalk [28] (http://mirwalk.umm.uni-heidelberg.de) and TargetScan (http://www.targetscan.org) to predict miRNAs that may bind to key immune markers. The predicted miRNA/mRNA binding axis was intersected with the miRNA/mRNA correlation axis estimated in the previous step to obtain the key miRNA/mRNA signal axis. The bioinformatics was used to draw the Venn diagram. The miRNA/mRNA signal axis binding sites were also plotted.

### 2.9. Gene Set Enrichment Analysis (GSEA)

In this step, we used the GSEA software [29] to perform Gene Ontology (GO) enrichment analysis, Kyoto Encyclopedia of Genes and Genomes (KEGG) enrichment analysis, and immunological characteristics (IC) analysis on the matrix data. The results with *p* < 0.05 were considered significant enrichment. The bioinformatics was used to draw the GO term plot, GSEA software was used to draw the GSEA enrichment plot in KEGG, and the ggplot2 [30] analysis package was used to draw the multiGSEA enrichment plot in IC.

### 2.10. Immune Infiltration Analysis

In this step, we used the CIBERSORT [15] analysis package to estimate the immune infiltration of the data set, and the expression matrix of immune infiltrating cells in different samples was obtained. Then, we used the estimate [31] analysis package to estimate the immune microenvironment of the transcriptome matrix, and the immune scores in different samples was obtained. The corrplot [32] analysis package was used to visualize the correlation between immune infiltrating cells involved in immune microenvironment.

### 2.11. Correlation Analysis between Key Immune Markers and Immune Infiltrating Cells

The tidyverse [33] analysis package and ggstatsplot [34] analysis package were used to analyze the correlation between the key immune markers and immune infiltrating cells. The correlation coefficient plot was generated. The results with *p* < 0.05 were considered statistically significant.

## 3. Results

### 3.1. Results of Data Processing Process

**Figure 1:**
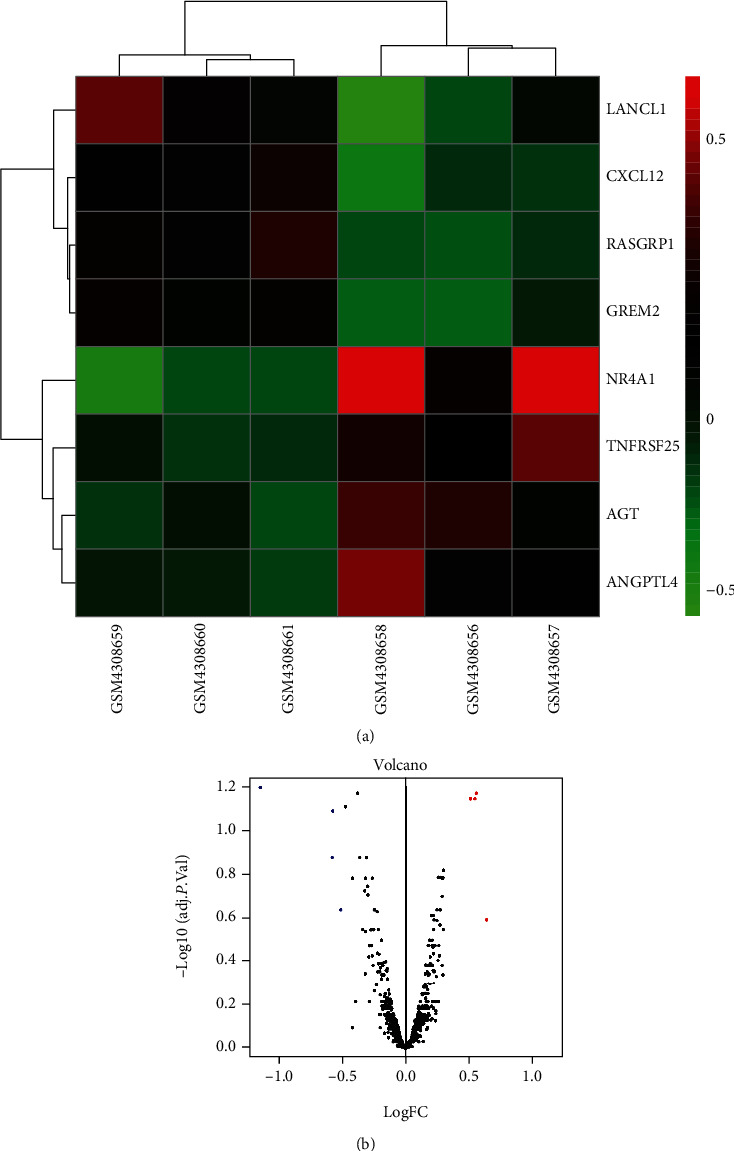
DEGs in the data set: (a) Heatmap of 8 immune DEGs; (b) volcano map of the immune genes; blue represents downregulated immune DEGs, and red represents upregulated immune DEGs.

**Figure 2:**
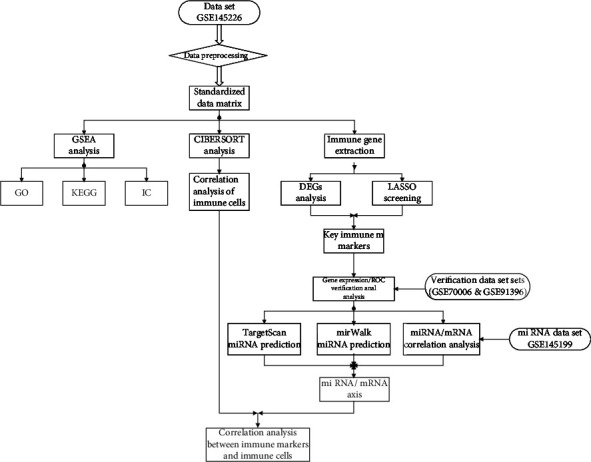
Flow chart of research analysis. GSEA: gene set enrichment analysis; GO: gene ontology; KEGG: Kyoto Encyclopedia of Genes and Genomes; IC: immunological characteristics; DEGs: differential expressed genes; LASSO: least absolute shrinkage and selection operator; ROC: receiver operating characteristic.

In this study, GSE145199 data set and GSE145226 data set were used for analysis. Firstly, a total of 976 immune genes were extracted from the transcriptome matrix data; secondly, 8 immune DEGs were obtained by differential gene analysis (Figure 1); thirdly, 21 hubgenes were screened by LASSO regression analysis. After crossing with immune DEGs, 1 key immune marker was obtained, namely, LANCL1; fourthly, after correlation analysis and online database prediction, a miRNA/mRNA axis was obtained, namely, miR-6325/LANCL1 axis, and included in the final analysis (Figure 2).

### 3.2. Results of LASSO Screening

**Figure 3:**
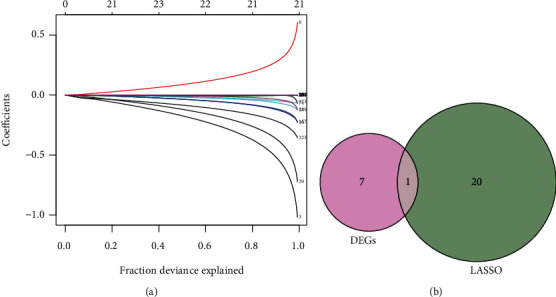
Results of hubgenes screening: (a) the fitted regression curve in LASSO model; (b) Venn diagram of hubgenes screening.

A total of 21 hubgenes were screened out by the LASSO model; the fitted regression curve was shown in Figure 3(a); after the intersection, 1 key immune marker was obtained, namely, LANCL1, as shown in Figure 3(b).

### 3.3. Results of ROC Verification in Key Immune Marker

**Figure 4:**
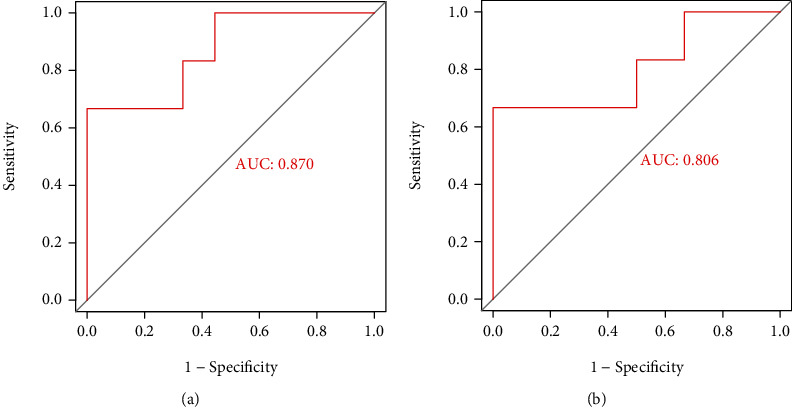
Results of ROC verification: (a) ROC curve of LANCL1 in verification data set of GSE70006; (b) ROC curve of LANCL1 in verification data set of GSE91396.

The ROC analysis results showed that LANCL1 has good predictability in GSE70006 (AUC = 0.870, *p* < 0.05) and GSE91396 (AUC = 0.806, *p* < 0.05) (Figure 4).

### 3.4. Results of Correlation Analysis and Online Database Prediction

**Figure 5:**
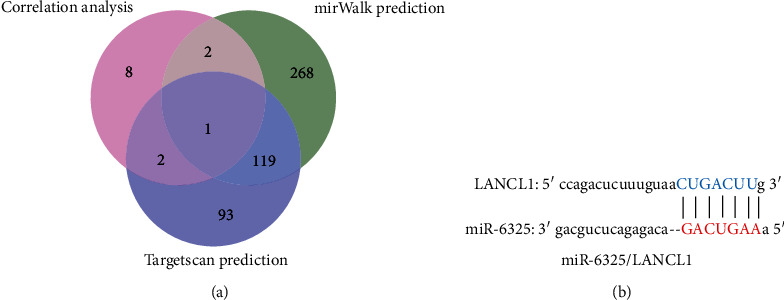
Results of correlation analysis and online database prediction: (a) Venn diagram of miRNA prediction; (b) the binding site of miR-6325/LANCL1 axis.

A total of 13 miRNAs were found to be negatively correlated with LANCL1. Meanwhile, mirWalk database predicted that 390 miRNAs might be bind to LANCL1. TargetScan database predicted that 215 miRNAs might be bind to LANCL1. After intersection, 1 miRNA/mRNA axis was obtained, namely, miR-6325/LANCL1 axis (Figure 5).

### 3.5. Results of GSEA Analysis

**Figure 6:**
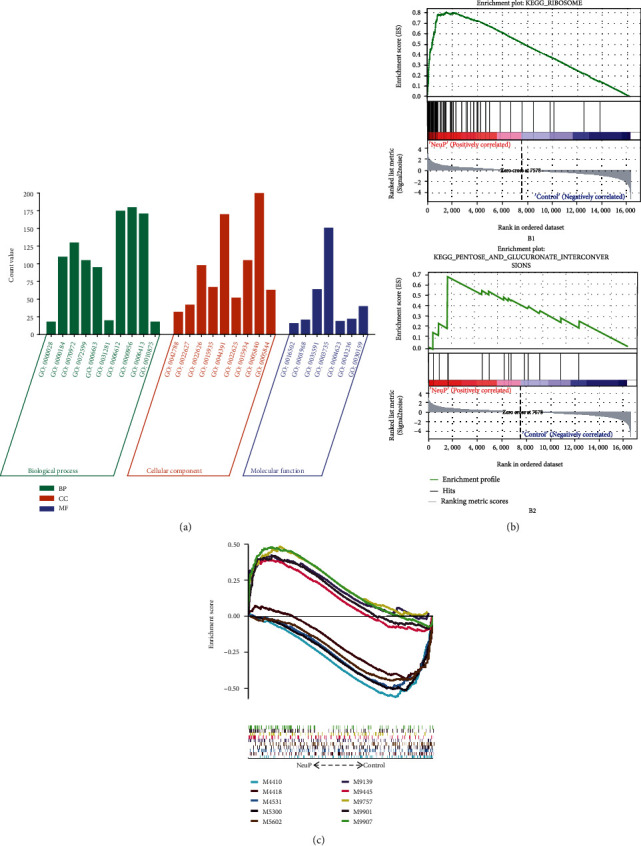
Results of GSEA enrichment analysis: (a) GO term plot of GO enrichment analysis; (b1) enrichment plot of ribosome pathway in KEGG analysis; (b2) enrichment plot of pentose and glucuronate interconversions pathway in KEGG analysis; (c) multiGSEA enrichment plot in IC analysis.

The results of GO enrichment analysis showed that NeuP genes were mainly related to ribosomal small subunit assembly, nuclear-transcribed mRNA catabolic process, nonsense-mediated decay, protein localization to endoplasmic reticulum, establishment of protein localization to endoplasmic reticulum, cotranslational protein targeting to membrane, positive regulation of cyclase activity, protein targeting to membrane, nuclear-transcribed mRNA catabolic process, translational initiation, and positive regulation of cholesterol efflux in biological process (BP); mainly related to polysomal ribosome, cytosolic small ribosomal subunit, cytosolic ribosome, small ribosomal subunit, ribosomal subunit, cytosolic large ribosomal subunit, large ribosomal subunit, ribosome, and polysome in cellular component (CC); and mainly related to nucleotide receptor activity, fibronectin binding, signaling adaptor activity, modified amino acid transmembrane transporter activity, structural constituent of ribosome, phospholipase A2 activity, laminin binding, and signaling receptor complex adaptor activity in molecular function (MF) (Figure 6(a)). The results of KEGG analysis showed that NeuP genes were mainly related to ribosome pathway (Figure 6(b1)) and pentose and glucuronate interconversions pathway (Figure 6(b2)). The results of IC analysis showed that NeuP genes were mainly related to CD4+ T regulatory cells functions, CD4+ T follicular helper cells functions, dendritic cells (DCs) functions, endogenous retroviruses (ERVs)-related immune response, naive and effector CD8+ T cells functions, and macrophages functions (Figure 6(c)).

### 3.6. Correlation Analysis Results between Genes and Immune Infiltrating Cells

**Figure 7:**
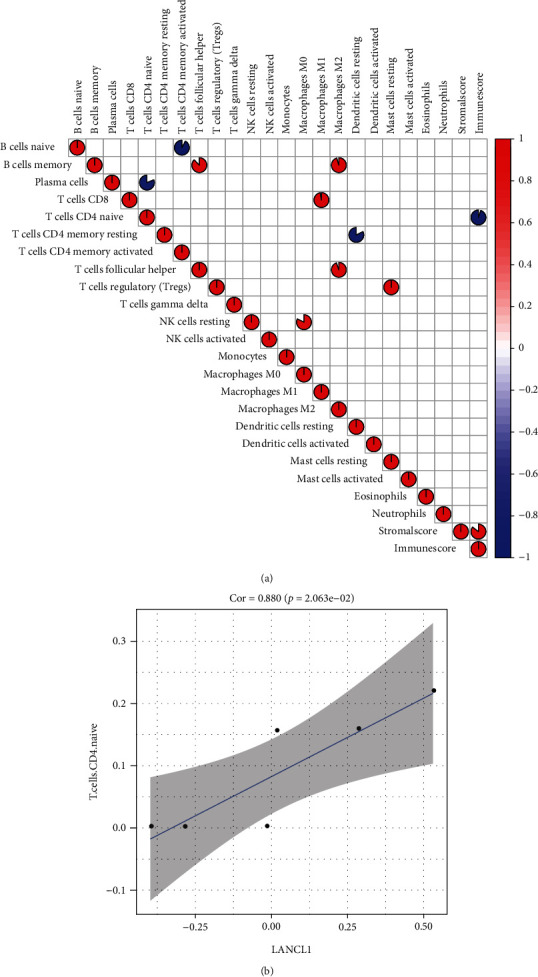
Correlation analysis results between genes and immune infiltrating cells: (a) correlation analysis of immune infiltrating cells; (b) correlation curve between LANCL1 and T cells CD4 naïve.

Correlation analysis results showed that B cells naïve was negatively correlated with T cells memory activated; B cells memory was positively correlated with T cells follicular helper and macrophages M2; plasma cells was negatively correlated with T cells CD4 naïve; T cells CD8 was positively correlated with macrophages M1; T cells CD4 naïve was negatively correlated with immune score; T cells CD4 memory resting was negatively correlated with dendritic cells resting; T cells follicular helper was positively correlated with macrophages M2; T cells regulatory (Tregs) was positively correlated with Mast cells resting; NK cells resting was positively correlated with macrophages M0 (Figure 7(a)). LANCL1 was positively correlated with T cells CD4 naïve (*r* = 0.880, *p* < 0.05) (Figure 7(b)).

## 4. Discussion

Because of the characteristics of the easily recurrent and difficult treatment of NeuP, it seriously affects the quality of life of the population. Therefore, it is very important to find out the mechanism of NeuP. At present, the mechanism of inflammatory response and the imbalance of immune microenvironment of NeuP has been paid more and more attention by researchers [35]. In this study, we screened the immune genes closely related to NeuP, and estimated the infiltrating degree of immune infiltrating cells in the immune microenvironment. In this study, we found LANCL1 as a key immune marker of NeuP and also found a miRNA/mRNA axis that closely related to LANCL1, namely, miR-6325/LANCL1 axis. We found the LANCL1 was positively correlated with T cells CD4 naïve. In addition, T cells, B cells, NK cells, macrophages, and dendritic cells have obvious correlation, suggesting the changes of immune microenvironment in NeuP.

In the IC analysis results, we found that the NeuP gene was mainly related the biological process of T cell and macrophages. In the results of immune infiltration analysis, we also found a positive correlation between T cells and macrophages. After peripheral nerve injury, an increase in the number of T cells has been found in the dorsal root ganglia and spinal cord, which suggests that they may play a role in NeuP [36]. After nerve injury occurs, mast cells will be activated first and release too much histamine [37] and TNF [38] and other cytokines, which will lead to nociceptor sensitivity and help the recruitment of neutrophils and macrophages. Both neutrophils and macrophages can produce and release cytokines such as TNF and prostaglandin E2 (PGE2) [39], which can further sensitize nociceptors. The aforementioned cellular activities promote the recruitment of T cells, and T cells can release a variety of cytokines according to their subtypes [40]. The recruitment of immune cells and the release of cytokines aggravate the inflammatory response of nerve injury, leading to NeuP. In our results, T cells, macrophages, and mast cells have an obvious positive correlation, which verifies the important role of the above-mentioned immune cascade in the mechanism of NeuP. The cascade reaction started by the activation of mast cells continuously recruits macrophages and T cells and releases excessive cytokines, leading to the continuous enhancement of the inflammatory response.

CD4+ T cells are helper cells among T cells, which are divided into two subtypes, namely, T helper 1 (Th1) and Th2 cells [41]. Previous studies have shown that Th1 cells produce interleukin-1 (IL-2) and interferon gamma (IFN-*γ*), which are involved in cell-mediated inflammation; Th2 cells produce IL-4, IL-6, IL-9, IL-10, and IL-13, which are involved in antibody and allergic reactions, and inhibit Th1 cells from synthesizing proinflammatory cytokines [42]. Our results showed that CD4 + T cells were negatively correlated with immune score, suggesting that some CD4 + T cells had protective effect on nerve injury. At the same time, our results also found that LANCL1 was positively correlated with CD4 + T cells, and the expression of LANCL1 was downregulated in NeuP, suggesting that LANCL1 may be a protective factor in the neuroinflammatory response, and LANCL1 may participate in the immune-related signal regulation process. Previous studies have found that LANCL1 has the function of resisting oxidative stress and protecting nerve cells [43, 44]. Decreased expression of LANCL1 affects the protective effect of related pathways on damaged nerves. Downregulation of the miR-6325/LANCL1 axis may be involved in the progression of NeuP. The role of miR-6325 is still unknown, but this gene has also been found in previous NeuP sequencing screening [45], suggesting that miR-6325 may be involved in some regulatory pathways of NeuP. Whether it is only involved in the miR-6325/LANCL1 axis still needs further exploration. Our results also found a negative correlation between B cells and CD4+ T cells. At present, the role of B cells in NeuP is still unclear and needs further exploration [8, 9].

## 5. Conclusion

In this study, we found that LANCL1 may be a protective factor for NeuP, and the miR-6325/LANCL1 axis may be involved in the occurrence and development of NeuP. Cascade reactions including mast cells, macrophages, and T cells may be an important reason for the aggravation of nerve damage.

## Data Availability

The sequencing data used to support the findings of this study have been deposited in the GEO repository (GSE145199, GSE145226, GSE70006, and GSE91396).
